# *N*′-(2-Chloro­benzyl­idene)-4-hydroxy­benzohydrazide

**DOI:** 10.1107/S1600536809030797

**Published:** 2009-08-08

**Authors:** Yu-Mei Hao

**Affiliations:** a Department of Chemistry, Baicheng Normal University, Baicheng 137000, People’s Republic of China

## Abstract

In the mol­ecule of the title compound, C_14_H_11_ClN_2_O_2_, the dihedral angle between the benzene rings is 30.53 (4)°. In the crystal structure, inter­molecular O—H⋯O and N—H⋯O hydrogen bonds link the mol­ecules into a two-dimensional network. π–π contacts between benzene rings [centroid–centroid distance = 3.619 (1) Å] may further stabilize the structure. The crystal studied was found to be an inversion twin.

## Related literature

For general background, see: Ali *et al.* (2008 ▶); Dao *et al.* (2000 ▶); Kargar *et al.* (2009 ▶); Karthikeyan *et al.* (2006 ▶); Sriram *et al.* (2006 ▶); Yeap *et al.* (2009 ▶). For related structures, see: Eltayeb *et al.* (2008 ▶); Fun *et al.* (2009 ▶); Hao (2009 ▶); Nadeem *et al.* (2009 ▶). For bond-length data, see: Allen *et al.* (1987 ▶).
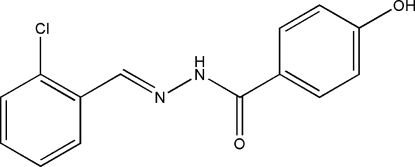


## Experimental

### 

#### Crystal data

C_14_H_11_ClN_2_O_2_
*M* *_r_* = 274.70
Orthorhombic, 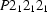

*a* = 7.2851 (17) Å
*b* = 11.716 (3) Å
*c* = 14.978 (3) Å
*V* = 1278.4 (5) Å^3^
*Z* = 4
Mo *K*α radiation
μ = 0.30 mm^−1^
*T* = 298 K
0.20 × 0.20 × 0.18 mm

#### Data collection

Bruker SMART CCD area-detector diffractometer
Absorption correction: multi-scan (*SADABS*; Sheldrick, 1996 ▶) *T*_min_ = 0.943, *T*_max_ = 0.948
6989 measured reflections
2360 independent reflections
1617 reflections with *I* > 2σ(*I*)
*R*_int_ = 0.045

#### Refinement

*R*[*F* ^2^ > 2σ(*F* ^2^)] = 0.046
*wR*(*F* ^2^) = 0.105
*S* = 1.02
2360 reflections
176 parameters
1 restraint
H atoms treated by a mixture of independent and constrained refinement
Δρ_max_ = 0.15 e Å^−3^
Δρ_min_ = −0.26 e Å^−3^
Absolute structure: Flack (1983 ▶), 963 Friedel pairs
Flack parameter: 0.45 (12)

### 

Data collection: *SMART* (Bruker, 2002 ▶); cell refinement: *SAINT* (Bruker, 2002 ▶); data reduction: *SAINT*; program(s) used to solve structure: *SHELXS97* (Sheldrick, 2008 ▶); program(s) used to refine structure: *SHELXL97* (Sheldrick, 2008 ▶); molecular graphics: *SHELXTL* (Sheldrick, 2008 ▶); software used to prepare material for publication: *SHELXL97*.

## Supplementary Material

Crystal structure: contains datablocks global, I. DOI: 10.1107/S1600536809030797/hk2750sup1.cif

Structure factors: contains datablocks I. DOI: 10.1107/S1600536809030797/hk2750Isup2.hkl

Additional supplementary materials:  crystallographic information; 3D view; checkCIF report

## Figures and Tables

**Table 1:** Hydrogen-bond geometry (Å, °)

*D*—H⋯*A*	*D*—H	H⋯*A*	*D*⋯*A*	*D*—H⋯*A*
O2—H2⋯O1^i^	0.82	1.84	2.657 (3)	179
N2—H2*A*⋯O2^ii^	0.90 (3)	2.106 (17)	2.951 (3)	157 (3)

Symmetry codes: (i) 
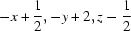
; (ii) 
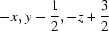
.

**Fig. 1.**
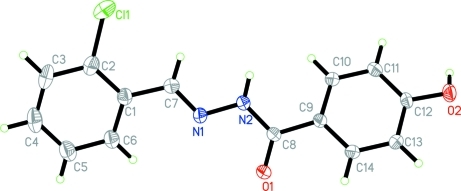
The molecular structure of the title molecule with the atom-numbering scheme. Displacement ellipsoids are drawn at the 30% probability level

**Fig. 2.**
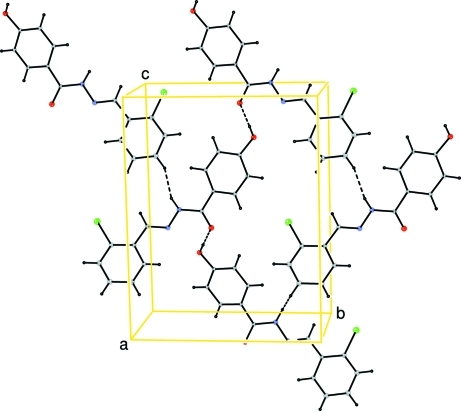
A partial packing diagram. Hydrogen bonds are shown as dashed lines.

## supplementary crystallographic information

### Comment

Schiff base compounds are a class of important materials used in pharmaceutical and medicinal fields (Dao *et al.*,  2000; Sriram *et al.*, 2006; Karthikeyan *et al.*,  2006). Schiff bases have also been used as versatile ligands in coordination chemistry (Ali *et al.*,  2008; Kargar *et al.*, 2009; Yeap *et al.*,  2009). Recently, the crystal structures of a large number of Schiff base compounds have been reported (Fun *et al.*,  2009; Nadeem *et al.*,  2009; Eltayeb *et al.*,  2008). As a part of our ongoing investigation (Hao, 2009), we report herein the crystal structure of the title new Schiff base compound.

In the molecule of the title compound (Fig. 1), the bond lengths (Allen *et al.*,  1987) and angles are within normal ranges. Rings A (C1-C6) and B (C9-C14) are, of course, planar and the dihedral angle between them is A/B = 30.53 (4)°.

In the crystal structure, intermolecular O-H···O and N-H···O hydrogen bonds (Table 1) link the molecules into a two-dimensional network (Fig. 2), in which they may be effective in the stabilization of the structure. The π–π contact between the benzene rings, Cg1—Cg2^i^ [symmetry code: (i) 1/2 + x, 1/2 - y, 1 - z, where Cg1 and Cg2 are centroids of the rings A (C1-C6) and B (C9-C14), respectively] may further stabilize the structure, with centroid-centroid distance of 3.619 (1) Å.

### Experimental

For the preparation of the title compound, 2-chlorobenzaldehyde (0.1 mmol, 14.1 mg) and 4-hydroxybenzohydrazide (0.1 mmol, 15.2 mg) were refluxed in a methanol solution (30 ml) for 30 min to give a clear orange solution. Yellow block-shaped single crystals of the compound were formed by slow evaporation of the solvent over several days at room temperature.

### Refinement

Atom H2A (for NH) was located in a difference Fourier map and refined as riding in as-found relative position, U_iso_(H) = 1.82U_eq_(N). The remaining H atoms were positioned geometrically with O-H = 0.82 Å (for OH) and C-H = 0.93 for aromatic H atoms, respectively, and constrained to ride on their parent atoms, with U_iso_(H) = xU_eq_(C,O), where x = 1.5 for OH H and x = 1.2 for aromatic H atoms.

### Crystal data

**Table:**

C_14_H_11_ClN_2_O_2_	*F*(000) = 568
*M**_r_* = 274.70	*D*_x_ = 1.427 Mg m^−^^3^
Orthorhombic, *P*2_1_2_1_2_1_	Mo *K*α radiation, λ = 0.71073 Å
Hall symbol: P 2ac 2ab	Cell parameters from 1016 reflections
*a* = 7.2851 (17) Å	θ = 2.4–24.5°
*b* = 11.716 (3) Å	µ = 0.30 mm^−^^1^
*c* = 14.978 (3) Å	*T* = 298 K
*V* = 1278.4 (5) Å^3^	Block, yellow
*Z* = 4	0.20 × 0.20 × 0.18 mm

### Data collection

**Table:**

Bruker SMART CCD area-detector diffractometer	2360 independent reflections
Radiation source: fine-focus sealed tube	1617 reflections with *I* > 2σ(*I*)
graphite	*R*_int_ = 0.045
ω scans	θ_max_ = 25.5°, θ_min_ = 2.2°
Absorption correction: multi-scan (*SADABS*; Sheldrick, 1996)	*h* = −8→8
*T*_min_ = 0.943, *T*_max_ = 0.948	*k* = −14→13
6989 measured reflections	*l* = −16→18

### Refinement

**Table:**

Refinement on *F*^2^	Secondary atom site location: difference Fourier map
Least-squares matrix: full	Hydrogen site location: inferred from neighbouring sites
*R*[*F*^2^ > 2σ(*F*^2^)] = 0.046	H atoms treated by a mixture of independent and constrained refinement
*wR*(*F*^2^) = 0.105	*w* = 1/[σ^2^(*F*_o_^2^) + (0.0459*P*)^2^ + 0.0042*P*] where *P* = (*F*_o_^2^ + 2*F*_c_^2^)/3
*S* = 1.02	(Δ/σ)_max_ < 0.001
2360 reflections	Δρ_max_ = 0.15 e Å^−^^3^
176 parameters	Δρ_min_ = −0.26 e Å^−^^3^
1 restraint	Absolute structure: Flack (1983), 963 Friedel pairs
Primary atom site location: structure-invariant direct methods	Flack parameter: 0.45 (12)

### Special details

**Table:**

Geometry. All esds (except the esd in the dihedral angle between two l.s. planes) are estimated using the full covariance matrix. The cell esds are taken into account individually in the estimation of esds in distances, angles and torsion angles; correlations between esds in cell parameters are only used when they are defined by crystal symmetry. An approximate (isotropic) treatment of cell esds is used for estimating esds involving l.s. planes.
Refinement. Refinement of F^2^ against ALL reflections. The weighted R-factor wR and goodness of fit S are based on F^2^, conventional R-factors R are based on F, with F set to zero for negative F^2^. The threshold expression of F^2^ > 2sigma(F^2^) is used only for calculating R-factors(gt) etc. and is not relevant to the choice of reflections for refinement. R-factors based on F^2^ are statistically about twice as large as those based on F, and R- factors based on ALL data will be even larger.

### Fractional atomic coordinates and isotropic or equivalent isotropic displacement parameters (Å^2^)

**Table:**

	*x*	*y*	*z*	*U*_iso_*/*U*_eq_	
Cl1	0.01997 (17)	0.34021 (7)	1.00869 (6)	0.0874 (4)	
O1	0.1624 (4)	0.92529 (17)	1.04651 (12)	0.0549 (6)	
O2	0.1212 (3)	1.16180 (18)	0.67205 (12)	0.0510 (6)	
H2	0.1881	1.1358	0.6330	0.077*	
N1	0.1278 (4)	0.69987 (19)	1.04360 (14)	0.0437 (7)	
N2	0.1146 (4)	0.7642 (2)	0.96676 (14)	0.0440 (7)	
C1	0.1105 (4)	0.5194 (2)	1.11448 (19)	0.0410 (7)	
C2	0.0682 (4)	0.4040 (3)	1.1104 (2)	0.0512 (9)	
C3	0.0627 (4)	0.3368 (3)	1.1861 (3)	0.0616 (10)	
H3	0.0335	0.2597	1.1817	0.074*	
C4	0.1006 (5)	0.3842 (3)	1.2681 (2)	0.0640 (10)	
H4	0.0949	0.3394	1.3192	0.077*	
C5	0.1472 (5)	0.4985 (3)	1.2746 (2)	0.0588 (10)	
H5	0.1749	0.5303	1.3298	0.071*	
C6	0.1523 (4)	0.5648 (3)	1.19849 (19)	0.0473 (8)	
H6	0.1841	0.6415	1.2032	0.057*	
C7	0.1054 (4)	0.5933 (2)	1.03614 (19)	0.0439 (8)	
H7	0.0855	0.5617	0.9800	0.053*	
C8	0.1310 (4)	0.8794 (2)	0.97441 (17)	0.0377 (7)	
C9	0.1139 (4)	0.9470 (2)	0.89145 (17)	0.0352 (7)	
C10	0.1551 (4)	0.9036 (2)	0.80756 (17)	0.0394 (7)	
H10	0.1814	0.8263	0.8012	0.047*	
C11	0.1576 (4)	0.9737 (2)	0.73349 (18)	0.0416 (8)	
H11	0.1876	0.9443	0.6777	0.050*	
C12	0.1154 (4)	1.0875 (2)	0.74280 (16)	0.0364 (7)	
C13	0.0671 (4)	1.1313 (2)	0.82502 (17)	0.0412 (7)	
H13	0.0335	1.2075	0.8306	0.049*	
C14	0.0692 (4)	1.0614 (2)	0.89873 (18)	0.0409 (8)	
H14	0.0401	1.0915	0.9544	0.049*	
H2A	0.074 (5)	0.729 (3)	0.9172 (14)	0.080*	

### Atomic displacement parameters (Å^2^)

**Table:**

	*U*^11^	*U*^22^	*U*^33^	*U*^12^	*U*^13^	*U*^23^
Cl1	0.1341 (10)	0.0427 (5)	0.0854 (7)	−0.0074 (6)	−0.0095 (7)	−0.0113 (5)
O1	0.0965 (19)	0.0376 (12)	0.0307 (11)	0.0005 (12)	−0.0119 (12)	−0.0021 (10)
O2	0.0687 (16)	0.0474 (13)	0.0370 (11)	0.0128 (13)	0.0090 (11)	0.0133 (10)
N1	0.0627 (18)	0.0340 (15)	0.0344 (13)	−0.0020 (13)	−0.0077 (14)	0.0041 (11)
N2	0.067 (2)	0.0337 (14)	0.0308 (13)	−0.0053 (13)	−0.0059 (15)	0.0026 (11)
C1	0.0396 (19)	0.0398 (18)	0.0435 (17)	0.0050 (15)	0.0051 (16)	0.0050 (14)
C2	0.054 (2)	0.0396 (19)	0.0604 (19)	0.0031 (15)	0.0022 (18)	0.0074 (16)
C3	0.052 (2)	0.043 (2)	0.089 (3)	−0.0012 (18)	0.008 (2)	0.026 (2)
C4	0.057 (2)	0.068 (3)	0.067 (2)	0.0149 (19)	0.011 (2)	0.0335 (19)
C5	0.064 (2)	0.065 (3)	0.048 (2)	0.011 (2)	0.0036 (19)	0.0125 (18)
C6	0.049 (2)	0.049 (2)	0.0434 (18)	0.0043 (16)	0.0009 (17)	0.0087 (16)
C7	0.056 (2)	0.0378 (18)	0.0378 (16)	0.0035 (16)	0.0009 (17)	−0.0018 (14)
C8	0.0477 (19)	0.0360 (16)	0.0294 (15)	−0.0026 (14)	−0.0012 (15)	0.0003 (12)
C9	0.0418 (18)	0.0320 (16)	0.0317 (14)	−0.0026 (13)	−0.0054 (15)	0.0005 (12)
C10	0.052 (2)	0.0340 (17)	0.0325 (15)	0.0014 (15)	0.0012 (15)	−0.0014 (13)
C11	0.052 (2)	0.0432 (19)	0.0297 (16)	0.0042 (16)	0.0012 (15)	−0.0009 (13)
C12	0.0416 (18)	0.0381 (17)	0.0295 (15)	0.0000 (15)	−0.0015 (15)	0.0083 (13)
C13	0.055 (2)	0.0309 (16)	0.0377 (16)	0.0052 (14)	0.0017 (15)	0.0004 (13)
C14	0.057 (2)	0.0345 (17)	0.0316 (15)	−0.0006 (14)	0.0032 (15)	−0.0025 (13)

### Geometric parameters (Å, °)

**Table:**

Cl1—C2	1.732 (3)	C5—C6	1.380 (4)
O1—C8	1.228 (3)	C5—H5	0.9300
O2—C12	1.372 (3)	C6—H6	0.9300
O2—H2	0.8200	C7—H7	0.9300
N1—N2	1.379 (3)	C8—C9	1.479 (4)
N1—C7	1.264 (3)	C9—C14	1.383 (4)
N2—C8	1.360 (3)	C9—C10	1.388 (4)
N2—H2A	0.90 (3)	C10—C11	1.381 (4)
C1—C2	1.388 (4)	C10—H10	0.9300
C1—C6	1.400 (4)	C11—C12	1.375 (4)
C1—C7	1.459 (4)	C11—H11	0.9300
C2—C3	1.381 (4)	C12—C13	1.380 (4)
C3—C4	1.376 (5)	C13—C14	1.375 (4)
C3—H3	0.9300	C13—H13	0.9300
C4—C5	1.384 (5)	C14—H14	0.9300
C4—H4	0.9300		
			
C12—O2—H2	109.5	N1—C7—H7	119.6
C7—N1—N2	117.2 (2)	C1—C7—H7	119.6
N1—N2—H2A	118 (2)	O1—C8—N2	121.7 (2)
C8—N2—N1	117.8 (2)	O1—C8—C9	121.4 (2)
C8—N2—H2A	123 (2)	N2—C8—C9	117.0 (2)
C2—C1—C6	117.3 (3)	C14—C9—C10	118.5 (2)
C2—C1—C7	122.5 (3)	C14—C9—C8	118.2 (2)
C6—C1—C7	120.2 (3)	C10—C9—C8	123.1 (3)
C3—C2—C1	121.7 (3)	C11—C10—C9	120.8 (3)
C3—C2—Cl1	118.0 (3)	C11—C10—H10	119.6
C1—C2—Cl1	120.3 (2)	C9—C10—H10	119.6
C4—C3—C2	119.8 (3)	C12—C11—C10	119.5 (2)
C4—C3—H3	120.1	C12—C11—H11	120.2
C2—C3—H3	120.1	C10—C11—H11	120.2
C3—C4—C5	120.2 (3)	O2—C12—C11	122.0 (2)
C3—C4—H4	119.9	O2—C12—C13	117.5 (2)
C5—C4—H4	119.9	C11—C12—C13	120.5 (2)
C6—C5—C4	119.6 (3)	C14—C13—C12	119.5 (3)
C6—C5—H5	120.2	C14—C13—H13	120.2
C4—C5—H5	120.2	C12—C13—H13	120.2
C5—C6—C1	121.5 (3)	C13—C14—C9	121.1 (3)
C5—C6—H6	119.2	C13—C14—H14	119.5
C1—C6—H6	119.2	C9—C14—H14	119.5
N1—C7—C1	120.8 (3)		

### Hydrogen-bond geometry (Å, °)

**Table:**

*D*—H···*A*	*D*—H	H···*A*	*D*···*A*	*D*—H···*A*
O2—H2···O1^i^	0.82	1.84	2.657 (3)	179
N2—H2A···O2^ii^	0.90 (3)	2.11 (2)	2.951 (3)	157 (3)

Symmetry codes: (i) −*x*+1/2, −*y*+2, *z*−1/2; (ii) −*x*, *y*−1/2, −*z*+3/2.
